# Short-Term Western Diet Aggravates Non-Alcoholic Fatty Liver Disease (NAFLD) With Portal Hypertension in TGR(mREN2)27 Rats

**DOI:** 10.3390/ijms21093308

**Published:** 2020-05-07

**Authors:** Carla Cremonese, Robert Schierwagen, Frank Erhard Uschner, Sandra Torres, Olaf Tyc, Cristina Ortiz, Martin Schulz, Alexander Queck, Glen Kristiansen, Michael Bader, Tilman Sauerbruch, Ralf Weiskirchen, Thomas Walther, Jonel Trebicka, Sabine Klein

**Affiliations:** 1 Department of Internal Medicine I, Goethe University Frankfurt, 60323 Frankfurt, Germany; Carla.Cremonese@kgu.de (C.C.); Robert.Schierwagen@kgu.de (R.S.); Frank.Uschner@kgu.de (F.E.U.); Sandra.Torres@kgu.de (S.T.); Olaf.Tyc@kgu.de (O.T.); Cristina.Ortiz@kgu.de (C.O.); Martin.Schulz@kgu.de (M.S.); Alexander.Queck@kgu.de (A.Q.); Sabine.Klein@kgu.de (S.K.); 2 Institute for Pathology, University of Bonn, 53127 Bonn, Germany; Glen.Kristiansen@ukbonn.de; 3 Max Delbrück Center for Molecular Medicine, 13092 Berlin, Germany; mbader@mdc-berlin.de; 4 Department of Internal Medicine I, University Hospital of Bonn, 53127 Bonn, Germany; sauerbruch@uni-bonn.de; 5 Institute of Molecular Pathobiochemistry, Experimental Gene Therapy and Clinical Chemistry (IFMPEGKC), RWTH University Hospital Aachen, 52074 Aachen, Germany; rweiskirchen@ukaachen.de; 6 Department of Pharmacology and Therapeutics, University College Cork, T12 YN60 Cork, Ireland; t.walther@ucc.ie; 7 Institute of Medical Biochemistry and Molecular Biology, University Medicine Greifswald, 17489 Greifswald, Germany; 8 Institute for Bioengineering of Catalonia, 08028 Barcelona, Spain; 9 European Foundation for the Study of Chronic Liver Failure, 08021 Barcelona, Spain; 10 Faculty of Health Sciences, University of Southern Denmark, 5000 Odense, Denmark

**Keywords:** ADGRE1, EMR1, F4/80, immunity, liver fibrosis, macrophage, NAFLD, portal hypertension, TGR(mREN2)27, Western diet

## Abstract

Non-alcoholic fatty liver disease (NAFLD) is gaining in importance and is linked to obesity. Especially, the development of fibrosis and portal hypertension in NAFLD patients requires treatment. Transgenic TGR(mREN2)27 rats overexpressing mouse renin spontaneously develop NAFLD with portal hypertension but without obesity. This study investigated the additional role of obesity in this model on the development of portal hypertension and fibrosis. Obesity was induced in twelve-week old TGR(mREN2)27 rats after receiving Western diet (WD) for two or four weeks. Liver fibrosis was assessed using standard techniques. Hepatic expression of transforming growth factor-β1 (TGF-β1), collagen type Iα1, α-smooth muscle actin, and the macrophage markers Emr1, as well as the chemoattractant Ccl2, interleukin-1β (IL1β) and tumor necrosis factor-α (TNFα) were analyzed. Assessment of portal and systemic hemodynamics was performed using the colored microsphere technique. As expected, WD induced obesity and liver fibrosis as confirmed by Sirius Red and Oil Red O staining. The expression of the monocyte-macrophage markers, Emr1, Ccl2, IL1β and TNFα were increased during feeding of WD, indicating infiltration of macrophages into the liver, even though this increase was statistically not significant for the EGF module-containing mucin-like receptor (Emr1) mRNA expression levels. Of note, portal pressure increased with the duration of WD compared to animals that received a normal chow. Besides obesity, WD feeding increased systemic vascular resistance reflecting systemic endothelial and splanchnic vascular dysfunction. We conclude that transgenic TGR(mREN2)27 rats are a suitable model to investigate NAFLD development with liver fibrosis and portal hypertension. Tendency towards elevated expression of Emr1 is associated with macrophage activity point to a significant role of macrophages in NAFLD pathogenesis, probably due to a shift of the renin–angiotensin system towards a higher activation of the classical pathway. The hepatic injury induced by WD in TGR(mREN2)27 rats is suitable to evaluate different stages of fibrosis and portal hypertension in NAFLD with obesity.

## 1. Introduction

Non-alcoholic fatty liver disease (NAFLD) has become one of the leading causes of cirrhosis in some countries due to the demographic change [1]. Especially fibrosis and development of portal hypertension have been shown to determine the necessity for treatment of NAFLD patients [2,3]. Development of portal hypertension is associated with morbidity and mortality in these patients [4,5]. Increased physical activity and reduction of weight are the current therapy strategies for NAFLD patients. If patients are able to lose five to ten percent of their weight, there is a chance to reduce hepatic fibrosis and inflammation, which is seen by an improvement in liver enzymes as well as reduced hepatic steatosis. However, many patients struggle with weight reduction and lifestyle change due to unhealthy dietary patterns and sedentary lifestyle [6], which emphasizes the need for adequate medical therapy, stopping the progress of fibrosis and portal hypertension in NAFLD.

The pathogenesis of NAFLD is complex and there are a number of drugs addressing NAFLD development, but not many that address fibrosis and portal hypertension [7]. One reason is the lack of suitable animal models, which are especially important for preclinical drug testing. In this study, we tried to create such a model using TGR(mREN2)27 rats after induction of obesity to investigate fibrosis and portal hypertension. The model of transgenic TGR(mREN2)27 rats, in which the mouse Ren-2 renin gene is inserted into the genome of the rat, was described in previous studies [8,9,10,11].

Activation of hepatic stellate cells (HSCs) leads to increased synthesis and deposition of collagen and other components of the extracellular matrix (ECM). Portal pressure is increased by the resulting stiffening of the liver and increased contractility of activated HSC [12]. In all these processes, the renin–angiotensin system (RAS) plays an important role, as renin is upregulated in human NASH patients. Higher levels of renin lead to an increased synthesis of angiotensin I and consequently angiotensin II, which is the agonist of the pro-fibrotic and pro-contractile AT1 receptor. This receptor facilitates fibrosis and portal hypertension through Janus kinase 2 (JAK2) in HSCs [13,14]. The role of angiotensin II induced AT1 receptor stimulation has already been demonstrated by Bataller et al. and supports our hypothesis [15,16].

In all these processes, macrophages originating from monocytic precursors have multiple functions. In particular, they are key regulators of liver repair, regeneration, and development of fibrosis. Particularly, the monocyte chemoattractant protein-1 (MCP-1), expressed by the gene Ccl2, is a key factor driving liver macrophage infiltration and steatosis [17]. Therefore, this chemokine has recently attracted much attention for the development of therapeutic strategies targeting NAFLD development [18,19,20]. In this regard, the EMR1, representing the mouse gene encoding the F4/80 antigen, has been proposed as a robust marker of liver-resident macrophages deriving from embryonic progenitors and as renewed independently of blood monocytes [21,22]. Since the pathophysiology of NAFLD is multifactorial and not yet completely understood, models for analyzing the impact of liver-resident macrophages (Kupffer cells) and infiltrating macrophages are urgently needed for analyzing the complex mechanisms of respective disease.

The overexpression of the mouse renin in TGR(mREN2)27 rats leads to chronic hypertension and spontaneous development of NAFLD with fibrosis and portal hypertension, but without obesity [10,11]. However, in humans obesity is strongly associated with hepatic steatosis [6]. Moreover, it was shown that obesity aggravates portal hypertension [23,24]. As obesity is strongly linked to NAFLD, the influence of obesity for the development of hepatic fibrosis and portal hypertension in NAFLD is an interesting research topic.

In this study, we analyzed the expression of Emr1 and Ccl2 during the onset of NAFLD. We aimed an aggravation of fibrosis and portal hypertension after induction of obesity receiving two and four weeks of Western diet (WD) in TGR(mREN2)27 rats. Our results indicate that this transgenic model is a suitable model to investigate aspects of NAFLD development when fed with the WD diet.

## 2. Results

### 2.1. Western Diet Induced Obesity in TGR(mREN2)27 Rats

**Figure 1:**
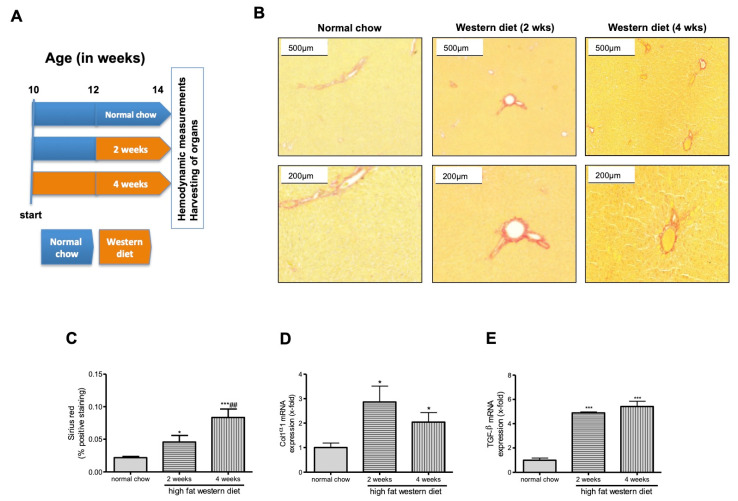
Fibrosis in transgenic TGR(mREN2)27 rats after high fat Western diet. (**A**) Ten-week-old rats received either normal chow for 4 weeks, or normal chow for 2 weeks followed by Western diet for 2 weeks or Western diet for 4 weeks before hemodynamic measurements and harvesting of the organs. (**B**) Hepatic Sirius Red staining show more collagen deposition in TGR(mREN2)27 rats receiving WD compared to normal chow. (**C**) The densitometric analysis of (**A**) shows an increase of collagen positive areas after 2 and 4 weeks of WD feeding. (**D**). Collagen 1α1 (Col1α1) mRNA expression quantified by RT-qPCR is increased in livers of TGR(mREN2)27 rats after 2 weeks of WD. After 4 weeks, a decrease in Col1α1 levels are noticed, still being higher compared to rats receiving normal chow. (**E**) Livers of TGR(mREN2)27 rats receiving 2 or 4 weeks of WD express significantly more tumor growth factor-β (TGF-β) than TGR(mREN2)27 rats fed with normal chow. */*** *p* < 0.05/0.01 vs. normal chow, ## *p* < 0.01 vs. 2 weeks high fat Western diet.

Two and four weeks of WD induced obesity in TGR(mREN2)27 rats (Figure 1A). In detail, rats receiving normal chow had a mean weight of 279 ± 13 g versus rats receiving WD for two weeks with a mean weight of 315 ± 7 g (*p* < 0.005) and rats receiving WD for four weeks with a mean weight of 341 ± 6 g (*p* < 0.005) (Table 1).

### 2.2. Western Diet Aggravated Liver Fibrosis in TGR(mREN2)27 Rats

Next, we analyzed, whether WD influenced liver fibrosis. The extent of fibrosis was assessed histologically in these animals. WD for two weeks increased the degree of liver fibrosis in TGR(mREN2)27 rats using Sirius Red staining (Figure 1B). This effect was even further aggravated after four weeks of WD, which was also observed in the quantification of hepatic Sirius red staining showing a significant difference compared to rats with normal chow (Figure 1C). This finding was confirmed by higher levels of collagen type I α1 (ColIα1) mRNA, however there was no further increase seen after four weeks of WD (Figure 1D). These results were also supported by the hepatic mRNA expression of fibrosis parameters like transforming growth factor-β (TGF-β) (Figure 1E).

**Figure 2:**
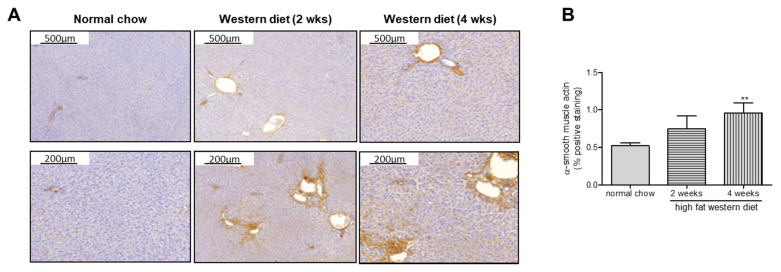
α-smooth muscle actin (α-SMA) expression in transgenic TGR(mREN2)27 rats after high fat Western diet. (**A**) Hepatic α-SMA staining increased after 2 and 4 weeks of WD (left panel). (**B**) This effect is also seen in the quantification of α-SMA staining by densitometry. ** *p* < 0.01 vs. normal chow.

Additionally, α-SMA immunohistochemistry revealed an increase with the duration of WD, confirmed by α-SMA quantification (Figure 2) as a marker of HSC activation.

### 2.3. Western Diet Induced Activation of Macrophages in TGR(mREN2)27 Rats

**Figure 3:**
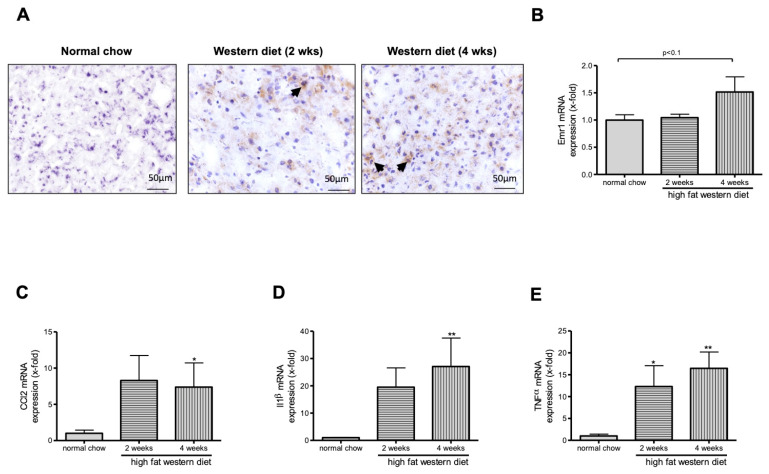
Hepatic assessment of macrophages and subgroup M1. (**A**) Hepatic frozen sections of TGR(mREN2)27 rats fed with normal chow, with 2 or 4 weeks of high fat Western diet were stained with the macrophage marker F4/80. Black arrow highlights positive cells. (**B**) Increased epidermal growth factor module-containing mucin-like receptor (Emr1) expression suggests increased numbers of macrophages after 4 weeks of WD feeding. (**C**) Increased expression of in CC-chemokine ligand 2 (Ccl2) was noticed after 4 weeks of WD feeding. (**D**,**E**) mRNA level of Il1β and TNFα confirmed the inflammatory effect after 2 and 4 weeks of WD feeding. */** *p* < 0.05/0.01 vs. normal chow.

F4/80 immunostaining suggest an increase of macrophages in TGR(mREN2)27 rats after WD feeding (Figure 3A). Interestingly, we could show a tendency towards M1 macrophage increase as shown by Emr1 expression (Figure 3B). Moreover, we observed an increase in Ccl2 (Figure 3C), Il1β (Figure 3D), and TNFα (Figure 3E) mRNA levels in TGR(mREN2)27 rats after WD diet compared to rats fed a normal chow.

### 2.4. Western Diet Induced NAFLD in TGR(mREN2)27 Rats

**Figure 4:**
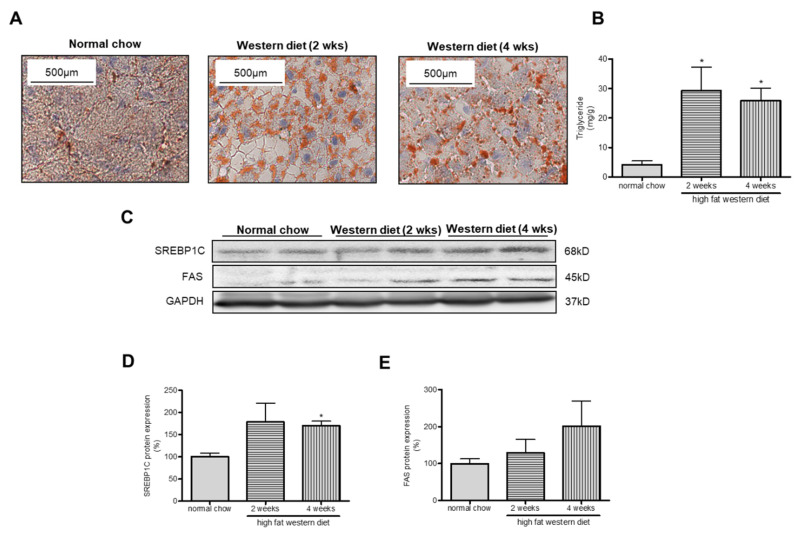
Steatosis assessment in TGR(mREN2)27 rats. Effect of 2 weeks and 4 weeks of high fat diet in liver steatosis in TGR(mREN2)27 rats. (**A**) Representative Oil Red O staining and polarized light imaging (magnification ×100) of livers from TGR(mREN2)27 rats fed with normal chow, 2 and 4 weeks of high fat diet. (**B**) Levels of hepatic triglyceride content in TGR(mREN2)27 rats fed with normal chow and with 2 or 4 weeks of high fat diet. (**C**) Western blots of steatosis marker proteins (sterol regulatory element-binding protein 1c (Srebp-1c), fatty acid synthase (FAS)) and their quantification (**D**,**E**). The scale bar is 100 µm. Results are represented as mean ± SEM. For comparisons a two-tailed student´s unpaired t-test was performed; controls, *n* = 5 normal chow TGR(mREN2)27; *n* = 5 TGR(mREN2)27 with 2 weeks of high fat diet; *n* = 5 TGR(mREN2)27 with 4 weeks of high fat diet. All analyses were carried out using GraphPad Prism. * *p* < 0.05 vs. TGR(mREN2)27 rats fed with normal chow.

As a proof for NAFLD development in TGR(mREN2)27 rats we demonstrated an increase in hepatic fat content and hepatic mRNA and protein expression of two key genes involved in lipogenesis; sterol regulatory element binding protein-1c (*SREBP-1c*) and fatty acid synthase (*FAS*) (Figure 4). The increase of *SREBP1C* levels was statistically significant after four weeks of WD while the increase of FAS was not significant. Induction of NAFLD was also seen in the quantifications of Oil Red O stainings and a significant increase in hepatic triglyceride content after two and four weeks of WD.

### 2.5. Western Diet Aggravated Portal Hypertension in TGR(mREN2)27 Rats

**Figure 5:**
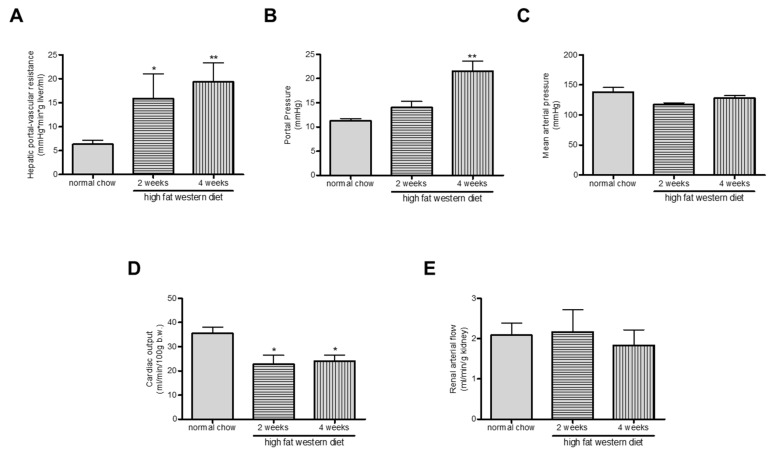
Hepatic and systemic effects of Western diet in TGR(mREN2)27 rats. (**A**) Portal pressure is increased after two and four weeks of WD. (**B**) Hepatic portal-vascular resistance increases after WD. (**C**) Mean arterial pressure shows no relevant alteration after WD feeding. (**D**) Cardiac output is reduced in rats receiving WD. (**E**) The renal arterial flow in TGR(mREN2)27 rats was investigated using the colored microsphere technique. Columns illustrate the renal arterial flow in mL/min/g kidney of rats. */** *p* < 0.05/0.01 vs. normal chow.

Since fibrosis was aggravated, we analyzed the magnitude of portal hypertension in TGR(mREN2)27 rats after WD. Interestingly, the additional WD in TGR(mREN2)27 rats aggravated portal hypertension known to have already a priori portal hypertension (Figure 5A), with a further increase after four weeks of WD. Hepatic-portal vascular resistance (HPVR) was higher in WD rats (Table 2, Figure 5B). In trend, mean arterial pressure was decreased in two and four weeks WD rats (Figure 5C) but did not reach significance. Cardiac output was decreased in TGR(mREN2)27 rats receiving two or four weeks of WD compared to the control group receiving normal chow (Figure 4D). The renal arterial flow in TGR(mREN2)27 rats was investigated using the colored microsphere technique. WD did not change renal arterial flow significantly (Figure 5E).

### 2.6. Western Diet and Role of RAS in TGR(mREN2)27 Rats

**Figure 6:**
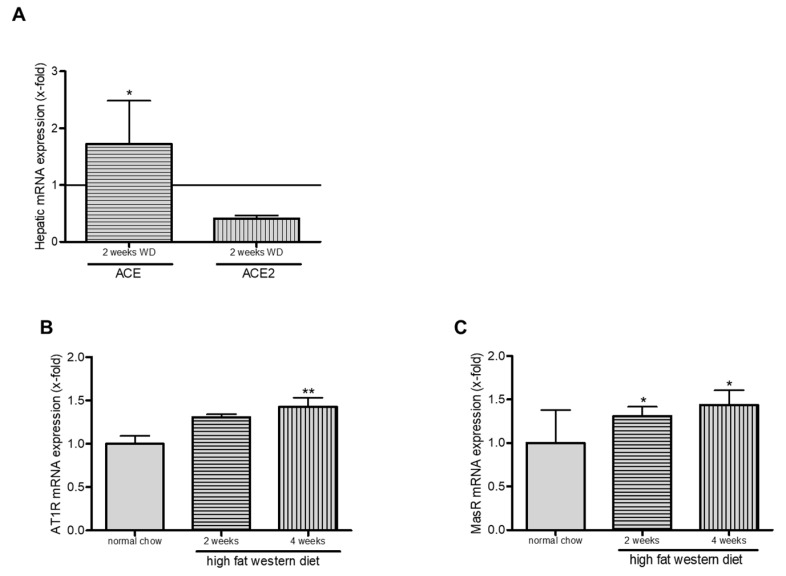
Western diet and RAS in TGR(mREN2)27 rats. Effect of high fat Western diet in RAS-system in TGR(mREN2)27 rats. (**A**) Increase in ACE and decrease in ACE2 levels. (**B**,**C**) Angiotensin-1-receptor and Mas-receptor were upregulated after two and four weeks of WD. */** *p* < 0.05/0.01 vs. normal chow.

We analyzed the role of RAS in TGR(mREN2)27 rats after WD. We could show an increase in ACE1 expression and decrease in ACE2 levels (Figure 6A). Angiotensin-1-receptor and Mas-receptor were increased significantly after four weeks of WD (Figure 6B,C).

## 3. Discussion

This study describes a modification of the liver pathology in the TGR(mREN2)27 rat model with induction of obesity, with relevant changes in macrophage infiltration, fibrosis and portal hypertension. We could show that WD induces obesity in this rat model and aggravates portal hypertension und fibrosis as well as an upregulation of fibrosis parameters, reaching significance especially for TGF-β mRNA. Moreover, expression levels of Emr1 and Ccl2 that, both activate macrophage recruitment, are increased during WD feeding.

NAFLD is an emerging etiology of liver disease. Portal hypertension is a frequent complication in NAFLD, especially in advanced disease stages [25]. It has been described elsewhere that TGR(mREN2)27 rats spontaneously develop pronounced steatosis and inflammation after twelve weeks of life [10]. Additionally, we could demonstrate that TGR(mREN2)27 rats also develop mild fibrosis and significant portal hypertension in the absence of any special diet [11] and might be used as a model for NAFLD research. The additional effect and role of obesity was not investigated in TGR(mREN2)27 rats so far, even though obesity is strongly linked to NAFLD. In NAFLD, arterial hypertension and obesity are both part of the metabolic syndrome. In our model we induced obesity by WD in TGR(mREN2)27 rats, which spontaneously show arterial hypertension. This is probably a different sequence of mechanism leading to NASH, having arterial hypertension as a first hit. It was previously shown that in NAFLD arterial hypertension was associated with progression of fibrosis [26,27]. Unfortunately, although arterial hypertension is a risk factor for progression of fibrosis, our animal models demonstrates that arterial hypertension as the first hit seems not to be a strong inductor of fibrosis but of portal hypertension.

Nevertheless the spontaneous development of NALFD in TGR(mREN2)27 rats overexpressing mouse renin underlines the pathogenic role of the renin-angiotensin system (RAS) in non-alcoholic liver disease. RAS upregulation activates a signaling cascade via angiotensin I and angiotensin II, which is the agonist of the pro-fibrotic and pro-contractile AT1 receptor. Stimulation of the AT1 receptor in HSC induces fibrosis and portal hypertension through Janus kinase 2 (JAK2) [13,14]. As a consequence, inhibition of renin leads to improved liver fibrosis and reduction in portal hypertension. Overexpression of angiotensin II induces NAFLD in TGR(mREN2)27 rats [10].

There are studies investigating the effects of obesity on portal hypertension and fibrosis in patients with cirrhosis, showing that obesity aggravates portal hypertension due to an upregulation of pro-inflammatory, pro-fibrogenic and pro-angiogenic processes [23,24]. These findings comply with the results of our study, showing that WD for two or four weeks aggravated fibrosis and portal hypertension in transgenic TGR(mREN2)27 rats.

We could show that TGR(mREN2)27 rats fed with WD show activation of macrophages, mainly M1. It is known that NAFLD as hepatic manifestation of metabolic disease is driven by chronic inflammatory processes in which macrophages play an important role. The polarization status of macrophages is influenced by metabolic stimuli such as fatty acids. There is increasing evidence from animal and clinical studies that macrophage targeting may be an effective therapeutic strategy for NAFLD [28]. Two macrophage populations are involved in chronic liver injury, the liver-resident Kupffer cells and the inflammatory monocyte-derived macrophages [29]. We could demonstrate a significant increase in gene expression of TGF-β and a relevant increase in Emr1 and Ccl2 mRNA levels, even though for Emr1 the increase was statistically not significant. TGF-ß, Emr1 and Ccl2 play an important role in the recruitment and activity of macrophages, which are key drivers in the pathogenesis of fibrosis. Emr1, originally named F4/80, is a mouse macrophage-restricted protein expressed in liver-resident Kupffer cells and in many macrophage populations [30,31]. Therefore, this surface protein is still the best marker to identify tissue macrophages [32]. An upregulation of Ccl2 expression preceded the increase of macrophages. MCP-1 is the main chemotactic factor necessary to recruit monocytes into foci of active inflammation and directly mediates a pro-fibrotic effect on fibroblasts by affecting TGF-β signaling. This in turn, stimulates synthesis and deposition of collagen [33]. In line, mice lacking the receptor for MCP-1 showed reduced infiltration and inflammatory macrophages in experimental fibrosis [34]. Therefore, the TGR(mREN2)27 rat model when fed with WD is a suitable NAFLD model that mimics key aspects of inflammation, fibrosis, portal hypertension and development of obesity.

The Baveno VI guideline acknowledge that co-factors of liver injury may maintain portal hypertension [35]. Surveillance for complications of portal hypertension such as variceal progress is stratified by co-factors such as obesity and alcohol abuse. In our earlier studies, we investigated the effect of an additional cholestatic or toxic liver injury in the model of TGR(mREN2)27 rats, not showing a relevant aggravation of portal hypertension and fibrosis, probably due to decreased expression of mouse renin in hepatocytes [11]. In this study, we could demonstrate that obesity increases portal hypertension in TGR(mREN2)27 rats, underlining the importance of this co-factor in surveillance of complications of portal hypertension. Spontaneous portal hypertension in TGR(mREN2)27 rats is mediated by increased portal pressure and hepatic vascular resistance [11]. When WD is admitted and obesity is induced the features of portal hypertension seem to be different. The detailed hemodynamic assessment of our study demonstrates that the hemodynamic effects were probably also mediated via extrahepatic components of the diet, possibly on the gut permeability mediating splanchnic hyperperfusion. This is in line with previous and recent results, demonstrating that diet and microbiome might play an important role in the development of fatty liver, mainly due to systemic or intestinal inflammation [36,37,38]. We failed to show an upregulated inflammation in these animals upon WD, and therefore assume that the additional hepatic effects of WD are independent of the liver phenotype.

Mouse and rat renin show a homology of 88% [39]. Mullins et al. showed that the introduction of the mouse Ren-2 renin gene into the genome of the rat caused arterial hypertension, demonstrating that the mouse renin gen is fully active in rats [8]. Our work demonstrates a shift towards higher AT1 receptor stimulation due to increased ACE expression and decreased ACE2 expression with increased AT1 and MAS receptor mRNA expression levels. This may indicate that the underlying mechanism is a shift towards the classical renin–angiotensin system pathway.

Our study has several limitations. Firstly, we used an artificial transgenic rat model with very little spontaneous steatosis. However, we know that renin is upregulated in human NASH patients similar to our TGR(mREN2)27 rat model. Secondly, we did not have a wild-type control group, however the research question was to analyze the additional effect of WD to RAS overexpression. Rats were only fed for maximum of four weeks with WD. A longer feeding period would be interesting to further analyze hypertension and fibrosis. However, prolongation of the feeding period with WD would lead to more severe illness due to uncontrolled arterial hypertension. Furthermore, we have only performed mRNA expression studies for Emr1, Ccl2, IL1β and TNFα. However, a previous study in kidney has shown that the TGR(mREN2)27 rats, developing angiotensin II-dependent hypertension, exhibited a more marked infiltration of macrophages than normotensive rats combined with elevated MCP-1/CCL2 expression during experimental inflammation [39]. Therefore, another limitation of this study is the lack of protein data for F4/80 and MCP-1 as well as CXCR3 levels.

In summary, our study shows that WD induces obesity in TGR(mREN2)27 rats and aggravates NAFLD, portal hypertension and fibrosis. In line, we observed an upregulation of profibrotic markers especially TGF-β and an activation of macrophages seen by increased Ccl2 and Emr1 mRNA expression. Probably the surplus on angiotensin II generation by increased ACE and AT1 receptor expression is a trigger for increased fibrosis and portal hypertension in our model. This may be also confirmed by previous studies, which show that AT1 receptor stimulation induces fibrosis and activation of hepatic stellate cells [15]. This may explain our findings. Therefore, this model is proposed as a suitable tool to analyze the pathogenic events of fibrosis and portal hypertension occurring during the pathogenesis of NAFLD.

## 4. Materials and Methods

### 4.1. Animals and Models of Liver Disease

For our experiments, we used five TGR(mREN2)27 rats per group (normal chow, WD for two weeks, WD for four weeks), giving a total number of 15 animals. It is known that in TGR(mREN2)27 rats NAFLD is established after twelve weeks of life [10]. Ten week old TGR(mREN2)27 rats received either normal chow for 4 weeks, or normal chow for 2 weeks followed by Western diet for 2 weeks or Western diet for 4 weeks before hemodynamic measurements and harvesting of the organs (Figure 1A). Experimental procedures were approved by the Animal Ethics Committee of North Rhine-Westphalia (approval code: LANUV 84-02.04.2014.A137; approval date: 28.05.2014). Rats were housed in individually ventilated cages with a12-hour light/12-hour dark cycle and temperature of 22 to 24 °C). Animals were fed standard rat chow ad libitum (Ssniff, Soest, Germany) with free access to water. For induction of obesity, TGR(mREN2)27 rats were fed for two or four weeks with WD ad libitum (20.85% fat, 19.5% casein, 1.25% cholesterol; Ssniff) as previously described [40] following standardized protocols [10,41]. The rationale to use WD is based on our previous work in which we induced NASH in another model [40].

### 4.2. Hemodynamic Studies

For the in-vivo hemodynamic studies, TGR(mREN2)27 rats were used after two and four weeks of WD as described previously [14,42] and compared to TGR(mREN2)27 rats receiving normal chow. To assess the effect of WD, mean arterial pressure and portal pressure were measured invasively. The colored microsphere technique was performed to investigate hemodynamics as described previously [14,42]. Briefly, 300,000 systemic (red/white) microspheres (15 µm diameter, Triton-Technologies, San Diego, CA, USA) were injected in the left ventricle and 150,000 microspheres (yellow/blue) were injected in the portal vein through the ileocecal vein to estimate the mesenteric portal-systemic shunt volume.

### 4.3. Tissue Collection

After the final experiment, animals were anesthetized and laparotomy was performed for tissue collection. The livers were cut into fragments and stored at −80 °C until further use as described previously [43,44]. Segments of each liver were fixed in formaldehyde (4%) for paraffin embedding, as described previously [13,44].

### 4.4. Sirius Red Staining and α-Smooth Muscle Actin Immunohistochemistry

Hepatic fibrosis was assessed in paraffin embedded liver sections (2–3 µm) stained with 0.1% Sirius Red in saturated picric acid (Chroma, Münster, Germany). Immunohistochemistry against α-smooth muscle actin (α-SMA) was performed in snap frozen liver samples. Cryosections (5–10 µm) of liver samples were fixed and incubated with mouse anti-α-SMA antibody (Sigma Aldrich, Munich, Germany) and a biotinylated donkey anti rat secondary antibody (Abcam, Cambridge, UK). Stained sections were digitalized using Pannoramic MIDI (3DHISTECH, Budapest, Hungary) and positive staining was quantified using Histoquant (3DHISTECH, Budapest, Hungary), as previously described [13,44].

### 4.5. Oil Red O Staining

Hepatic steatosis was assessed using Oil Red O stainings and performed as described previously [40,45]. Stainings were captured with a Nikon Digital Sight DS-Vi1 (Chiyoda, Tokyo, Japan). A minimum of 10 high-power fields was captured for analysis.

### 4.6. F4/80 Staining

Macrophage staining of livers were assessed in slides of frozen sections (5–10 µm). Slides were fixed in ice cold ethanol for 15–20 min. Slides were air-dried for 10 min and washed in PBS 2 times for 2 min. Thereafter, slides were quenched in 0.3% H_2_O_2_ for 10 min. PBS washing was repeated for 2 min and air dried. Then, 1–2 drops of Avidin solution (Abcam, Cambridge, UK) were added on each slide and incubated for 10 min. The washing procedure was repeated for 2 min. Next, 1–2 drops of Biotin blocking solution (Abcam) were added to each slide and incubated for 10 min at room temperature. After 2 times of washing in PBS, slides were incubated with 10% FCS for 30 min. Afterwards the diluted anti-F4/80 antibody (Merck, Taufkirchen, Germany) was given to slides and incubated for 2 hours. The slides were washed twice in PBS for 4 min and air dried. The secondary antibody was added to the slides and incubated for 1 hour. After washing the slides three times in PBS and air drying, the ABC reagent was added for 30 min before washing the slides three times with PBS. DAB solution (Vector Laboratories, Burlingame, CA, USA) was added to the slides and incubated for 5 min. Slides were washed and stained in hematoxylin for 5 min and washed two times for 2 min. Slides were air-dried before mounting.

### 4.7. Hepatic Western Blots

Protein levels were analyzed through Western blot analyses as described previously [45]. Briefly, snap-frozen livers were homogenized and diluted. Protein content of homogenates was determined with the DC assay kit (Bio-Rad, Munich, Germany). Samples (30 µg of protein/lane) were subjected to SDS-PAGE (10% gels), and proteins were blotted on nitrocellulose membranes. Glyceraldehyde-3-phosphate dehydrogenase served as an endogenous control (sc166545HRP, Santa Cruz Biotechnology, Santa Cruz, CA). The membranes were blocked and incubated with primary antibodies for either fatty acid synthase (ab82419, Abcam, Berlin, Germany), or sterol regulatory element-binding protein 1c (ab28481, Abcam, Berlin, Germany). Finally, membranes were incubated with the corresponding secondary antibody, and blots were developed using enhanced chemiluminescence. Protein quantification was performed by Multi Gauge V3.0 software (Fujifilm, Tokyo, Japan).

### 4.8. Hepatic Triglyceride Content

The hepatic triglyceride concentration was analyzed in snap-frozen liver samples using the colorimetric assay kit (ab65336, Abcam, Berlin, Germany) according to manufacturer’s instruction and protocol. 

### 4.9. Quantitative Real-Time PCR

Liver homogenates were prepared using previously described methods [13,44]. RNA isolation, reverse transcription, and detection of amplicons by real-time polymerase chain reaction were performed as described previously [13,44]. Briefly, RNA was isolated from samples using the Qiazol reagent (Qiagen, Hilden, Germany) as instructed by the manufacturer. The following predesigned TaqMan gene expression assays (Applied Biosystems, Foster City, CA, USA) were used: Col1a1 (Rn00801649_g1), Tgfb1 (Rn00572010_m1), Ccl2 (Rn00580555_m1), Emr1 (Rn01527631_m1), Il1b (Rn00580432_m1), and Tnfa (Rn01525859_g1). Samples were normalized to the content of 18S rRNA.

### 4.10. Statistical Analysis

Graphs are presented as means ± standard error of mean (SEM) with a group size of n = 5 per group. p-values < 0.05 were considered statistically significant. For gene expression experiments, fold-change was calculated by 2-ΔΔCT method and normalized to the respective control group [46]. Visualization of results and statistical analysis were performed using GraphPad Prism version 5.00 for Windows (GraphPad Software, La Jolla, CA, USA). The Mann–Whitney U test was used to test for statistical significant differences between groups.

## Figures and Tables

**Table 1:** Body weight, liver weight and spleen weight of rats receiving normal chow, 2 weeks and 4 weeks high fat Western diet.

Rat treatment	Body Weight (g)	Liver Weight (g)	Spleen Weight (g)
Normal chow	279.3 ± 13.2	11.4 ± 2.3	0.6 ± 0.7
High fat Western diet (2 wks)	315.4 ± 6.7 ***	12.1 ± 1.4	0.6 ± 0.2
High fat Western diet (4 wks)	341.0 ± 6.4 ***^#^	13.7 ± 1.7 *	0.6 ± 0.1

*/*** *p* < 0.05/0.001 vs. normal chow, ^#^ *p* < 0.05 vs. 2 weeks high fat Western diet.

**Table 2:** Hemodynamics in transgenic TGR(mREN2)27 rats with normal chow vs. 2 and 4 weeks of Western diet.

TGR(mREN2)27 Groups	Portal Pressure (mmHg)	Hepatic-Vascular Resistance (mmHg*min*g Liver/mL)	Splanchnic-Vascular Resistance (mmHg*min*100g/mL)	Mean Arterial Pressure (mmHg)	Systemic-Vascular Resistance (mmHg*min*100g/mL)
**normal chow**	11.24 ± 0.53	6.31 ± 0.83	49.92 ± 11.12	129.73 ± 6.68	4.68 ± 0.89
**2 weeks Western diet**	14.00 ± 1.29 *	15.91 ± 5.11 *	23.05 ± 4.42 *	117.40 ± 2.91	5.54 ± 0.65
**4 weeks Western diet**	21.50 ± 2.18 **^##^	19.36 ± 3.94 **	23.48 ± 4.70 *	127.80 ± 4.49	5.57 ± 0.75

*/** *p* < 0.05/*p* < 0.01 vs. TGR(mRen2)27 with normal chow; ^##^ *p* < 0.01 vs. TGR(mRen2)27 after 2 weeks of Western diet.

## Abbreviations

EMR1	EGF module-containing mucin-like receptor
FAS	Fatty acid synthase
IL1β	Interleukin 1-β
MCP-1	Monocyte chemoattractant protein-1
NAFLD	Non-alcoholic fatty liver disease
RAS	Renin-angiotensin system
α-SMA	α-smooth muscle actin
SREBP-1c	Sterol regulatory element binding protein-1c
TGF-β	Transforming growth factor-β
TNFα	Tumor necrosis factor α
WD	Western diet
